# The Efficacy of Eye Movement Desensitization and Reprocessing in Children and Adults Who Have Experienced Complex Childhood Trauma: A Systematic Review of Randomized Controlled Trials

**DOI:** 10.3389/fpsyg.2018.00534

**Published:** 2018-04-11

**Authors:** Runsen Chen, Amy Gillespie, Yanhui Zhao, Yingjun Xi, Yanping Ren, Loyola McLean

**Affiliations:** ^1^Beijing Key Laboratory of Mental Disorders, National Clinical Research Center for Mental Disorders, Beijing Anding Hospital, Capital Medical University, Beijing, China; ^2^The Clinical Psychology Center, Beijing Anding Hospital, Capital Medical University, Beijing, China; ^3^Department of Psychiatry, University of Oxford, Oxford, United Kingdom; ^4^Department of Orthodontics, Shanghai Engineering Research Center of Tooth Restoration and Regeneration, School and Hospital of Stomatology, Tongji University, Shanghai, China; ^5^Brain and Mind Centre and Discipline of Psychiatry, Sydney Medical School, University of Sydney, Sydney, NSW, Australia; ^6^Westmead Psychotherapy Program for Complex Traumatic Disorders, Discipline of Psychiatry, Sydney Medical School, University of Sydney and Western Sydney Local Health District, Parramatta, NSW, Australia; ^7^Consultation-Liaison Psychiatry, Royal North Shore Hospital, Sydney, NSW, Australia

**Keywords:** childhood trauma, EMDR, systematic review, PTSD symptoms, children and adult, complex trauma

## Abstract

**Background:** Survivors of complex childhood trauma (CT) such as sexual abuse show poorer outcomes compared to single event trauma survivors. A growing number of studies investigate Eye Movement Desensitization and Reprocessing (EMDR) treatment for posttraumatic stress disorder (PTSD), but no systematic reviews have focused on EMDR treatment for CT as an intervention for both adults and children. This study therefore systematically reviewed all randomized controlled trials (RCTs) evaluating the effect of EMDR on PTSD symptoms in adults and children exposed to CT.

**Methods:** Databases including PubMed, Web of Science, and PsycINFO were searched in October 2017. Randomized controlled trials which recruited adult and children with experience of CT, which compared EMDR to alternative treatments or control conditions, and which measured PTSD symptoms were included. Study methodology quality was evaluated with Platinum Standard scale.

**Results:** Six eligible RCTs of 251 participants were included in this systematic review. The results indicated that EMDR was associated with reductions in PTSD symptoms, depression and/or anxiety both post-treatment and at follow-up compared with all other alternative therapies (cognitive behavior therapy, individual/group therapy and fluoxetine) and control treatment (pill placebo, active listening, EMDR delayed treatment, and treatment as usual). However, studies suffered from significant heterogeneity in study populations, length of EMDR treatment, length of follow-up, comparison groups, and outcome measures. One study had a high risk of bias.

**Discussion:** This systematic review suggests that there is growing evidence to support the clinical efficacy of EMDR in treating CT in both children and adults. However, conclusions are limited by the small number of heterogenous trials. Further RCTs with standardized methodologies, as well as studies addressing real world challenges in treating CT are required.

## Introduction

Complex childhood trauma (CT) encompasses severe traumatic events that are likely to be chronic, disrupt personality development and lead to less trust in fundamental relationships (Kliethermes et al., 2014), as well as impacting upon neurological development (Ford and Courtois, 2009). Examples of complex childhood trauma are physical abuse, parental neglect, sexual abuse, emotional abuse along with domestic and community violence occurring in circumstances where escape is impossible.

Experiences of CT are unfortunately relatively common. An American study by Turner et al. (2010) found that 22% of a nationally representative sample of 2,030 participants aged 2 to 17 years reported exposure to four or more different types of trauma in their lifetime, including exposure to domestic violence, child abuse and war. A similar telephone survey study (Finkelhor et al., 2015) of 4,000 children aged 0–17 years old, also found that 15.2% had experienced maltreatment by the caregiver (including 5% who had experienced physical abuse), 5.8% had witnessed domestic violence between parents and 2% of girls had experienced sexual abuse. In one of the largest studies of adverse childhood experiences with over 9,000 participants, Felitti et al. (1998) found that a quarter reporting more than two categories of childhood trauma (including psychological, physical or sexual abuse, violence, and household dysfunction (living with household members who were substance abusers, mental disorder or suicidal or ever imprisoned). Focusing on sexual abuse, a Canadian telephone survey of 804 adults (Hébert et al., 2009) found that the prevalence of child sexual abuse was 22.1% for women and 9.7% for men, and a recent Australian study of 3,739 participants found even higher rates: at age 21, 19.3% of males and 30.6% of females reported that they had been exposed to sexual abuse in childhood (Mills et al., 2016).

Furthermore, some of the above studies have found that exposure to CT is associated with health risks. For example, Felitti et al. (1998) found a strong relationship between the number of CT exposures and the number of risk factors for leading causes of death in adults. DSM-IV field trials conducted on 400 traumatized patients and 128 community residents have also demonstrated that those exposed to chronic, repetitive childhood trauma, particularly at an early age, consistently presented with extensive symptoms including emotional deregulation, impulsivity, deficit of attention or consciousness, negative self-perception, self-destructive behavior, dissociation, and altered perceptions of the perpetrator (idealization, preoccupation with the perpetrator; adoption of the perpetrator's belief system), interpersonal difficulties and somatization (van der Kolk et al., 2005). They also reported that individuals who have experienced abuse during childhood show more dysregulation, more functional limitations related to interpersonal relationships and emotion control, and poorer treatment outcomes, than those who have been abused only during adulthood.

In particular, PTSD is prevalent among individuals who have experienced CT. In a nationally representative sample of American adults, those who had experienced CT were six times more likely to develop PTSD compared with adults who had not experienced CT (Afifi et al., 2009). Similarly, the Canadian telephone survey described above (Hébert et al., 2009) found that adults who were exposed to sexual abuse in childhood were likely to be currently experiencing psychological distress and PTSD symptoms.

One approach to treating CT-associated PTSD symptoms has been trauma-focused cognitive behavioral therapy (CBT), but high drop-out rates have been reported and some have suggested that prolonged exposure and a focus on trauma reprocessing is not appropriate (McDonagh et al., 2005; Cloitre et al., 2011; Boterhoven de Haan et al., 2017). This has led to exploration of alternative trauma-focused approaches for the treatment of CT.

Eye Movement Desensitization and Reprocessing (EMDR) is a structured and integrated psychotherapy combining different well-established psychotherapeutic techniques (such as imagined exposure, resource development, cognitive change, and self-control) with bilateral sensory stimulation, according to the dual focus of attention principle (Shapiro et al., 2007). The aim of EMDR is to facilitate information processing of emotionally distressing traumatic events with an 8-phase program: (1) history and treatment planning; (2) preparation; (3) assessment; (4) reprocessing and desensitization; (5) installation; (6) body scan; (7) closure; (8) re-evaluation of past, present, and future (Shapiro et al., 2007). Importantly, EMDR does not require intensive prolonged exposure to the traumatic experiences (Rogers and Silver, 2002). The efficacy of EMDR for PTSD in trauma survivors in adults (Seidler and Wagner, 2006; Chen et al., 2014, 2015; Cusack et al., 2016) and children and adolescents (Rodenburg et al., 2009; Gillies et al., 2013; Brown et al., 2017; Moreno-Alcázar et al., 2017) has been established in several systematic review and meta-analysis, but these studies have predominantly included participants who have experienced a single event trauma (such as natural or man-made disasters, physical assault, and traffic accident) (Meiser-Stedman et al., 2007) or have samples with heterogenous traumas.

Two systematic reviews have been conducted to examine the efficacy of psychological interventions for treating psychological distresses associated with childhood abuse. One reported that psychological interventions successfully reduced PTSD symptom severity in survivors of childhood sexual abuse (effect size: *g* = 0.72) (Taylor and Harvey, 2010), however the study did not focus on PTSD symptoms and not all treatments were PTSD-focused. Ehring et al. (2014) addressed these issues in a meta-analysis of trauma-focused interventions vs. non-trauma-focused interventions for PTSD symptoms in adult survivors of childhood abuse. The study concluded that trauma-focused interventions [including EMDR and Trauma-Focused Cognitive Behavior Therapy (TF-CBT)] were more efficacious than non-trauma-focused interventions, however, this meta-analysis only included adult survivors of childhood abuse. In doing so, it only included three studies on EMDR as an intervention for PTSD in survivors of CT. Additionally, it drew conclusions only on trauma-focused interventions as a heterogenous category.

Research indicates that children who have been abused benefit from treatment delivered as early as possible (Racco and Vis, 2015); the longer they are left to deal with traumatic memories, the less likely they are to make a full recovery, and the more likely they are to develop severe mental disorders in future. Therefore, the present review aimed to systematically summarize the current RCT evidence evaluating the effectiveness of EMDR interventions on PTSD symptoms in children, adolescent and adult survivors of CT.

## Methods

### Search

This study was performed in accordance with the preferred reporting items for systematic reviews and meta-analyses statement (PRISMA) (Moher et al., 2009). The studies in this systematic review were identified in three ways. Firstly, the authors searched a range of electronic databases including Web of Science (7th October 2017), PsycINFO (8th October 2017), PubMed (7th October 2017), and The Francine Shapiro Library (8th October 2017), for studies published at any time until the search date. The databases were searched using the following keywords: Eye Movement Desensitization and Reprocessing /EMDR and childhood trauma, complex trauma, multiple trauma, complex PTSD, abuse, neglect, attachment, maltreatment, interpersonal, dissociative, torture, war and violence. Secondly, the reference lists of the published systematic reviews were screened to identify other papers not appearing within the primary search. Finally, the authors searched the website “ClinicalTrials.gov” to identify any ongoing research studies. We contacted the authors of any registered clinical trials for further information and authors of eligible studies where there were a mix of children and young people requesting the respective proportions of the age sample.

### Criteria of eligibility

Studies complying with the following predefined criteria were included in this systematic review: (1) randomized controlled trial design; (2) sample included only participants (children, adolescents or adults) who had experienced complex childhood trauma/childhood abuse, including sexual abuse, physical abuse, maltreatment, torture, and violence; (3) studies compared EMDR interventions to control groups or an alternative intervention; (4) PTSD symptom severity was a treatment target with pre and post-treatment scores provided; (5) the full text of each identified study was available. Any study that did not meet any of these criteria was excluded. Two review authors (CR and ZY) independently screened the output of the search to identify potentially eligible studies. Duplicates were removed. All disagreements were resolved by consensus. The kappa score for inter-rater reliability was 0.86, which indicated good agreement between the reviewers in selection of the eligible studies.

### Extraction of data and outcomes of interest

Two reviewers independently extracted the data from included manuscripts using a standard extraction sheet. Any discrepancies were resolved by consensus between the reviewers. Relevant information for each study was extracted including the author's name, publication year, number of patients, age, gender, study completers of EMDR group, traumatic event, PTSD instrument, additional outcome variables, duration of follow-up, the type of control group-control (e.g., treatment as usual) or alternative psychological treatment (e.g., cognitive behavior therapy) and number of EMDR sessions. The primary outcome of interest was PTSD symptoms and the secondary outcomes of interest were depression and anxiety; we only report findings related to these outcomes.

### Quality of assessment

For all included studies, authors assessed the quality of studies using the Platinum Standard, which was specifically designed to evaluate effectiveness in EMDR research (Hertlein and Ricci, 2004). The Platinum Standard contains 13 comprehensive criteria for assessing research, including clearly defined target symptoms, reliable and valid measures, use of blind evaluators, information regarding an assessor's training, manualized, replicable, and specific treatment, random assignment, treatment adherence, non-confounded conditions, use of multimodal measures, length of treatment, level of therapist training, use of a control group, and effect size reporting. The authors rated the risk of bias descriptively for each criterion. Scores range between 0 and 13, with higher scores indicating higher quality.

## Results

### Search results

**Figure 1:**
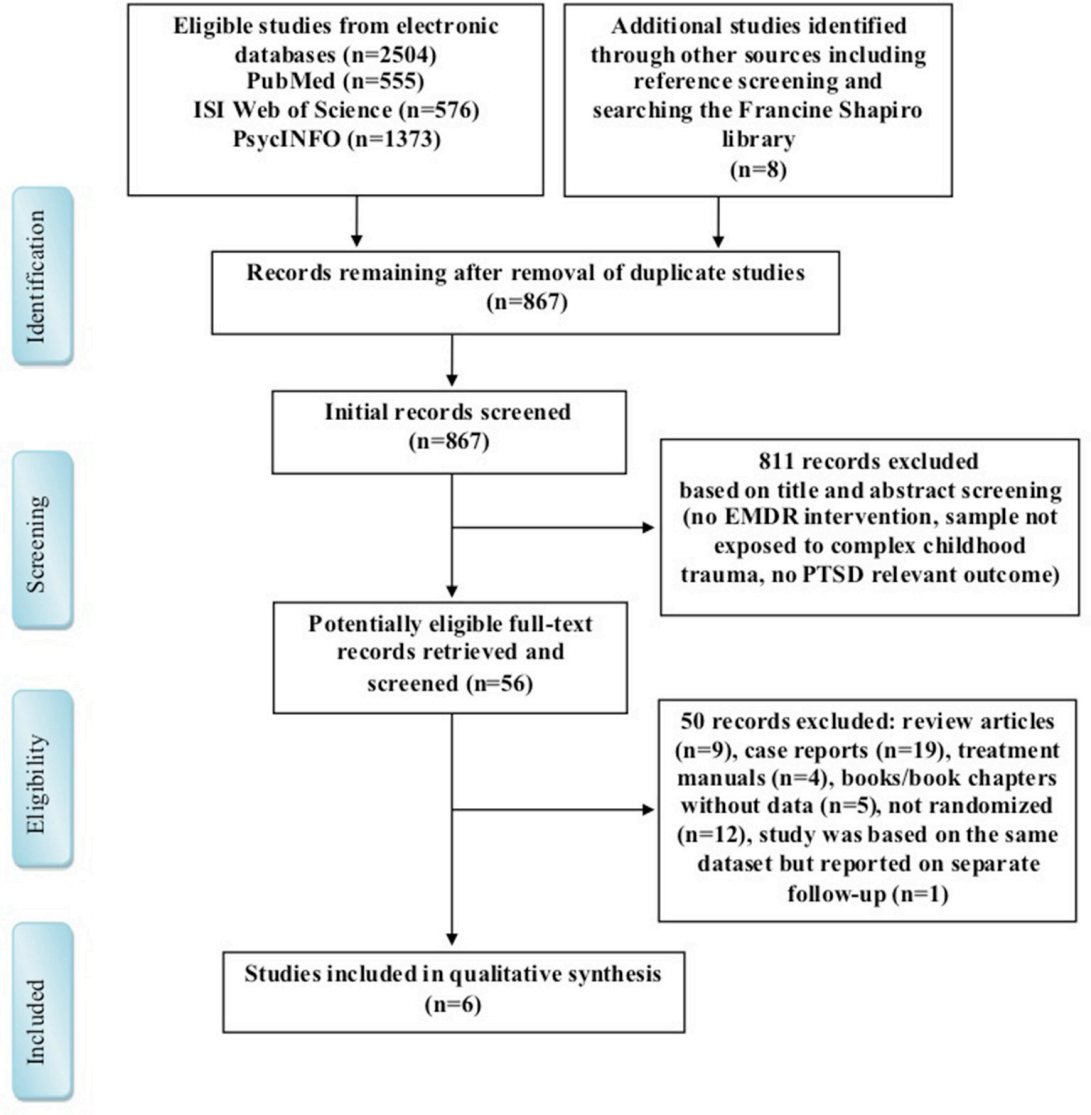
Preferred reporting items for systematic reviews and meta-analysis (PRISMA) flow diagram for search strategy and supply selection process.

Two thousand five hundred twelve publications were identified by the search strategy. Of these, we found 2,504 studies in the electronic databases (Web of Science, PubMed and PsycINFO database searches), while additional 8 publications were from references of identified studies or other sources (including The Francine Shapiro Library^1^). After removal of 867 duplicate studies, those not fulfilling the inclusion criteria and clearly irrelevant studies, we assessed 56 potential publications for full-text eligibility. In total 6 randomized controlled clinical trials were included in this systematic review (Scheck et al., 1998; Edmond et al., 1999; Soberman et al., 2002; Jaberghaderi et al., 2004; van der Kolk et al., 2007; Farkas et al., 2010). 2 studies reported EMDR vs. two other treatments (Edmond et al., 1999); each comparison is reported separately, therefore 8 comparisons between EMDR and an alternative are reported. Figure 1 shows the process of study selection.

### Characteristics of included studies (see Table 1)

A total of 231 participants were enrolled (*N* = 103 individuals treated with EMDR), with participant age ranging between 12 and 36. Three studies reported the outcomes of EMDR treatment in adolescents, while three reported on adult patients who were over 18 years old. Four comparisons evaluated the efficacy of EMDR vs. alternative interventions (including individual and group therapy, CBT, and fluoxetine) while four comparisons evaluated the efficacy of EMDR vs. control treatment groups (including placebo, active listening, delayed treatment, and treatment as usual). The number of EMDR sessions varied between 2 to 12. Due to the large heterogeneity of these studies, the existing information did not allow for a meta-analysis; the authors therefore report a qualitative review.

**Table 1:** Study characteristics of included studies.

**Study**	**Type of control group**	**Participant N EMDR/Control**	**Traumatic event**	**Adults/Children age**	**Female**	**Number of EMDR sessions**	**Time of follow-up assessments**	**PTSD measures**	**Additional outcome measures**	**PS score**
Edmond et al., 1999	Routine individual treatment	20/20	Sexual abuse	Adultslatha 18–35	100%	6	Post-treatmentlatha 3 months	IES	Depression-BDI Anxiety-STAI	9
	Delayed EMDR treatment	20/19								
Jaberghaderi et al., 2004	CBT	7/7	Sexual abuse	Childrenlatha 12–13	100%	4–8	2 weeks post-treatment	CROPS PROPS	N/A	8.5
Farkas et al., 2010	Routine individual/ group therapy	19/21	Maltreatment	Childrenlatha Mean = 14.6	62.5%	12	Post-treatment 3 months	DISC-PTSD symptoms TSCC-dissociation	Depression-TSCC Anxiety-TSCC	9
Scheck et al., 1998	Active listening	30/30	Mixed childhood abuse	Adultslatha 16-25	100%	2	Post-treatment 3 months	IES PENN	Depression-BDI Anxiety-STATE	9
Soberman et al., 2002	Treatment as usual	14/15	Multiple childhood traumas	Childrenlatha 10–16 (mean 13.35)	0	3	Post-treatment 2 months	IES CROPS PROPS	N/A	6.5
van der Kolk et al., 2007	Pill Placebo	11/14	Sexual and physical abuse	Adultslatha 18–65 (mean 36.1)	83%	8	Post-treatment 6 months	CAPS	N/A	10
	Fluoxetine	11/10								

### Risk of bias

The scores for risk of bias are shown in Table 2. The major shortcomings of enrolled studies were the lack of non-confounded conditions (item #8), and length of treatment (item #10). All the studies included reliable and valid measures (item #2). Furthermore, bias associated with level of therapist training (item #11) was also infrequent in the quality assessment. Except for one study demonstrated to have more risk of bias on study quality (Soberman et al., 2002), all the studies achieved scores ranging from 8.5 to 10.

**Table 2:** Platinum Standard (PS) scores for EMDR studies.

**Study**	**PS**	**PS**	**PS**	**PS**	**PS**	**PS**	**PS**	**PS**	**PS**	**PS**	**PS**	**PS**	**PS**	**Total**
	**#1**	**#2**	**#3**	**#4**	**#5**	**#6**	**#7**	**#8**	**#9**	**#10**	**#11**	**#12**	**#13**	
Edmond et al., 1999	0.5	1	0.5	0	1.0	1.0	1.0	1.0	0	0	1.0	1.0	1.0	9
Jaberghaderi et al., 2004	0.5	1	1	0.5	0.5	0.5	0.5	0	1.0	0.5	1.0	0.5	1.0	8.5
Farkas et al., 2010	0.5	1	1	1	0.5	1.0	0.5	0	1.0	1.0	1.0	0.5	0	9
Scheck et al., 1998	0.5	1.0	1.0	1.0	1.0	1.0	0.5	0	0.5	0	1.0	0.5	1.0	9
Soberman et al., 2002	1	1	0.5	0	1.0	0.5	0.5	0	1.0	0	0.5	0.5	0	6.5
van der Kolk et al., 2007	1	1	1	0.5	1.0	1.0	0.5	0	0.5	0.5	1.0	1.0	1.0	10

*EMDR, Eye movement desensitization and reprocessing; #1 clearly defined target symptoms (0, no clear diagnosis or symptom definition; 0.5, not all participants meet target symptom criteria; 1.0, all participants met target symptom criteria); #2 reliable and valid measures (0, did not use reliable and valid measures; 0.5, measures used inadequate to measure change; 1.0, reliable, valid, and adequate measures used); #3 use of blind evaluators (0, assessor was therapist; 0.5, assessor was not blind; 1.0, assessor was blind and independent); #4 information regarding an assessor's training (no training in administration of instruments used in the study); #5 manualized, replicable and specific treatment (0, treatment was not replicable or specific; 0.5, treatment replicable and specific but not standard EMDR protocol; 1.0, treatment followed EMDR training manual),; #6 random assignment (0, assignment not randomized; 0.5, only one therapist or other semi-randomized designs; 1.0, unbiased assignment to treatment); #7 treatment adherence (0, treatment fidelity poor; 0.5, treatment fidelity variable or self-monitored by therapist only; 1.0, treatment fidelity independently checked and adequate); #8 non-confounded conditions [0, most participants exposed to confounds with no control for variables; 1.0, confounds nonexistent or controlled for (e.g., exclusion, matched assignment, etc.)]; #9 use of multimodal measures (0, self-report measures only; 0.5, self-report plus interview or physiological or behavioral measures; 1, self-report plus two or more other types of measures); #10 length of treatment (0, 1–6 sessions; 0.5, 7–10 sessions; 1.0, 11+sessions); #11 level of therapist training (0, no qualifications for treating clinicians provided; 0.5, qualifications for treatment group, clinicians provided; 1.0, qualifications for treatment and comparative group, clinicians provided); #12 use of a control group (0, no use of a wait control/comparison group; 0.5, use of a comparison group but no control; a.0, use of a no-treatment control group); #13 effect size reporting (0, no effect size reported; 1.0, effect size reported)*.

### Synthesized findings (see Table 3)

#### EMDR in children exposed to CT

Three studies investigated the use of EMDR in children exposed to complex childhood trauma. Two of these exclusively included adolescents with conduct disorder (Soberman et al., 2002; Farkas et al., 2010). Soberman et al. (2002) studied 29 male adolescents with conduct disorder who had been exposed to unspecified multiple traumas. Participants received either 3 sessions of EMDR treatment or treatment as usual, and were then assessed at post-treatment and 2-month follow-up. The measures of PTSD symptoms were Impact of Event Scale (IES), The Child Report of Posttraumatic Symptoms (CROPS), and the Parent Report of Posttraumatic Symptoms (PROPS). Farkas et al. (2010) also studied 40 adolescents with conduct problems (all under youth protective services due to maltreatment); however, this sample was predominantly female, and participants received different interventions: either EMDR combined with motivation-adaptive skills-trauma resolution (MASTR) treatment or routine individual or group therapy. The measures of PTSD symptoms in this study were the Diagnostic Interview Schedule for Children (DISC) and Trauma Symptom Checklist for Children (TSCC), which were assessed post-treatment and at 3 months follow up.

**Table 3:** Results from included studies for measures of PTSD symptoms.

**Study**	**Comparison**	**IES (means)**	**CROPS (means)**	**PROPS (means)**	**CAPS (means)**	**PENN (means)**	**DISC-PTSD symptoms**	**TSCC-dissociation**
Edmond et al., 1999	Routine individual treatment	Post-treatment EMDR 14.1 vs. 14 Follow-up EMDR 10.3 vs. 18 Effect size 0.56						
	Delayed EMDR	Post-treatment EMDR 14.1 vs. 32.1^*^						
Jaberghaderi et al., 2004	CBT		Post-treatment EMDR 18.86 vs. 22.71	Post-treatment EMDR 10.14 vs. 11.29				
Farkas et al., 2010	Routine individual/ group therapy						Post-treatment EMDR 0.3 vs. 1.4^*^ Follow-up EMDR 0.4 vs. 1.3	Post-treatment EMDR 2.2 vs. 4.6^*^ Follow-up EMDR 2.9 vs. 3.3
Scheck et al., 1998	Active listening	Post-treatment EMDR 23.43 vs. 36.41^*^ Effect size 0.83 Follow-up EMDR 15.88 vs. 26.16				Post-treatment EMDR 20.36 vs. 31.22^*^ Effect size 0.72		
Soberman et al., 2002	Treatment as usual	Post-treatment (mean change in scores) EMDR −5.5 vs. −5.73 Follow-up EMDR −12.83 vs. −6.78	Post-treatment (mean change in scores) EMDR −4 vs. −3.36 Follow-up EMDR −8.83 vs. −4	Post-treatment (mean change in scores) EMDR −5.40 vs. −0.73^*^ Follow-up EMDR −2.33 vs. −2				
van der Kolk et al., 2007	Pill Placebo				Post-treatment EMDR 38.36 vs. 46.57^*^			
	Fluoxetine				Post-treatment EMDR 38.36 vs. 40.20 Follow up EMDR 33 vs. 50.43			

* *indicate statistically significant findings*.

Soberman et al. reported significantly greater decreases in PROPS scores post-test, and a trend for greater decreases in IES scores at 2-month follow up, in participants who had received EMDR in comparison to the treatment-as-usual group, however no other differences were statistically significant. In contrast, Farkas et al. report more consistent findings across the DISC and TSCC measures of significantly greater decreases in PTSD symptoms post-test and at 3-month follow-up, as well as significantly greater decreases in depression and anxiety scores post-test, in participants who had received EMDR in comparison to the routine therapy control group. In addition, the participants who received EMDR had no diagnoses of PTSD and no clinically relevant scores on depression, anxiety or dissociation measures post-treatment, and no diagnoses of PTSD or clinically relevant anxiety scores at follow-up. However, as EMDR was combined with MASTR in this study, it is difficult to determine whether these effects were caused specifically by the EMDR component of the study.

The third study in children (Jaberghaderi et al., 2004) reported findings for 14 Iranian girls ages 12–13 years who had been sexually abused and were randomized to receive either EMDR or CBT treatment. The number of sessions in each condition varied, and was determined by participants' self-reported decreases in distress. The CROPS and PROPS measures were used to assess participants 2 weeks post-treatment. There were no statistically significant differences between the groups, likely due to a lack of statistical power. However, EMDR showed greater effect sizes for reductions in symptoms on CROPS and PROPS and the authors commented that EMDR showed greater treatment efficiency, with participants allocated to EMDR requiring a mean of 6.1 sessions vs. participants allocated to CBT requiring a mean of 11.6 sessions to achieve similar results.

#### EMDR in adults exposed to CT

The other three studies investigated the use of EMDR in adults who had been exposed to complex childhood trauma. Two of these looked exclusively at women (Scheck et al., 1998; Edmond et al., 1999). Edmond et al. studied 59 adult female survivors of childhood sexual abuse aged 18–35; the majority had experienced prolonged and repeated abuse, abuse at the hands of multiple perpetrators, and additional physical abuse and adult revictimization. Participants were randomly assigned to one of the three groups: 6 sessions of EMDR treatment, routine individual treatment, or delayed EMDR treatment group. PTSD symptoms were measured using the IES post-treatment and at 3 months follow up; in addition, the State-Trait Anxiety Inventory (STATE) and Beck Depression Inventory (BDI) were used to measure anxiety and depression. Scheck et al studied 60 young women aged 16–25 who had experienced multiple forms of childhood trauma (90% had experienced physical or sexual abuse) and had recently shown dysfunctional behavior. They were allocated to receive either two 90-min. sessions of EMDR or active listening, and the same measures (IES, STATE, BDI) were used, as well as the Penn Inventory for Posttraumatic Stress Disorders (PENN), post-treatment and at 3 months.

Edmond et al. found that at post-treatment measurement, EMDR was significantly better at reducing IES scores than control (delayed treatment), but there was no significant difference between EMDR and routine individual therapy. However, at 3-month follow-up, there was a (non-significant) moderate effect size for the difference between EMDR and routine individual therapy for IES scores and significantly greater decreases in depression and anxiety scores for participants who had received EMDR compared to those who had received routine individual therapy. Furthermore, a 2004 report of an 18-month follow-up of 42/59 participants in this sample (Edmond and Rubin, 2004) found that these benefits were maintained. Similarly, Scheck et al found significantly greater improvements on all measures for the EMDR group compared to the active listening group post-treatment, with moderate to large effect sizes and sustained improvements to follow-up despite only two sessions.

Finally, one study compared EMDR to drug treatment (van der Kolk et al., 2007). This study included 35 adult participants aged 18–65 who had been exposed to childhood trauma of sexual and physical abuse and had current diagnoses of PTSD. Participants were allocated to receive either 8 weeks treatment with EMDR, fluoxetine or a placebo pill. They were assessed post-treatment and 6-month follow-up using the Clinician-Administered PTSD Scale (CAPS), DSM-IV version. Post-treatment, participants who had received EMDR treatment showed lower CAPS scores than those who had received fluoxetine or placebo and a higher rate of remission than those on placebo, and at follow-up showed lower CAPS scores and higher rate of remission than those allocated fluoxetine treatment; however, none of these differences were statistically significant.

#### Post-treatment scores vs. follow-up scores

Of the five studies with follow-up data two studies found that, at follow-up, the EMDR group showed greater improvements than post-treatment while the comparison groups showed deterioration (Edmond et al., 1999; van der Kolk et al., 2007); two studies found that all groups showed greater improvements at follow-up (Scheck et al., 1998; Soberman et al., 2002); and one study found that the EMDR group deteriorated while the control group improved (Farkas et al., 2010).

#### Measures of PTSD symptoms

Three studies used the IES to measure PTSD symptoms (Scheck et al., 1998; Edmond et al., 1999; Soberman et al., 2002), two of which found significant results. Two studies used the CROPS measure and neither found significant results (Soberman et al., 2002; Jaberghaderi et al., 2004). All other measures that were used found at least one significant difference.

#### Comparison groups

When EMDR was compared to control groups such as delayed treatment, active listening, treatment-as-usual or placebo, all studies reported at least some significant differences (Scheck et al., 1998; Edmond et al., 1999; Soberman et al., 2002; van der Kolk et al., 2007). When EMDR was compared to CBT, no significant differences were reported, though greater effect sizes and greater treatment efficiency were reported (Jaberghaderi et al., 2004). When EMDR was compared to non-specific individual or group therapy, one study reported significant differences (Farkas et al., 2010) and another reported no significant differences (Edmond et al., 1999). When EMDR was compared to drug treatment, no significant differences were reported, though a greater number of participants no longer met criteria for PTSD at 6-month follow up (van der Kolk et al., 2007).

#### Drop-out

Across all 6 studies, none found a significant difference in drop out numbers between EMDR groups and comparison groups.

## Discussion

The present study is the first to systematically evaluate RCTs in both adults and children investigating the effectiveness of EMDR in treating PTSD symptoms associated with exposure to complex childhood trauma. All six included studies demonstrated favorable outcomes for both children and adults allocated to EMDR in comparison to non-specific therapy, CBT, fluoxetine, and control conditions (delayed EMDR treatment, active listening, treatment as usual and pill placebo), albeit with variable consistency and some differences not reaching statistical significance. In addition, three studies measured symptoms of depression and anxiety symptoms and the results consistently indicated greater reductions in the EMDR group (Scheck et al., 1998; Edmond et al., 1999; Farkas et al., 2010).

EMDR may have several benefits for dealing with patients who have experienced CT, many of which were noted within the included studies. Patients are offered a great deal of control over the whole treatment process, including the ability to choose the time and level of exposure to aversive inner experiences, such as feelings, thoughts and mental images. These can be experienced in short bursts rather than through the prolonged instances experienced in exposure therapy. The unique reprocessing technique in EMDR allows patients with chronic trauma to avoid verbalizing their trauma, which may facilitate the desensitization and processing of the aversive memories (Korn, 2009). We also found a low drop-out rate for all included studies, with an average completion rate of 95.5% in the EMDR group, which is consistent with other reports of EMDR dropout rates below 10% (Marcus et al., 1997; Ironson et al., 2002); this is in contrast with 41% dropout rates reported in studies of CBT for childhood trauma (McDonagh et al., 2005). Interestingly, all of the studies in this systematic review were individual interventions rather than group interventions, which is consistent with previous meta-analytic findings reporting that individual sessions yield larger effect sizes than group treatment for adult survivors of childhood abuse (Taylor and Harvey, 2010; Ehring et al., 2014).

It is noteworthy that currently there is no consensus on the number of EMDR sessions recommended to treat CT and in this review, we found studies with interventions varying from two sessions to twelve. Some authors suggest at least 12 or 20 sessions are needed to achieve more lasting improvement beyond PTSD symptom reduction (Carlson et al., 1998; Brown and Shapiro, 2006), though it likely depends on the extent and complexity of the trauma and an individual's complex set of protective and risk factors. Adolescents and adults who experience trauma over longer timescales are likely to require longer sessions of EMDR treatment to resolve the psychological changes associated with trauma (van der Kolk et al., 2007). However, one meta-analysis of EMDR in children (Rodenburg et al., 2009) found that fewer sessions (3.5 average) were associated with better treatment outcomes and in the present systematic review, one study with just two sessions reported some of the most consistent significant improvements (Scheck et al., 1998). Due to the lack of consensus on the number of EMDR sessions required for patients who've experienced CT, future studies should report and discuss this information.

This review highlights the limited studies investigating EMDR as an intervention for children who have experienced CT, with no RCTs investigating pre-adolescent children, and future research will need to address this thoughtfully. It has been suggested that before the age of 12, children exposed to CT experience posttraumatic symptoms without full PTSD syndromes: developmental regression, dysregulation of sleep, disruptive behavior, refusal to attend school, disorganized attachment and increased aggressive behavior (Sheet, 2001; Petersen et al., 2014). Children who do not necessarily meet sufficient diagnostic criteria for the diagnosis of PTSD may still be symptomatic and impaired (Cohen and Scheeringa, 2009). However, children aged 12–18 who have been exposed to CT tend to present with symptoms which more closely match the diagnostic criteria presented in the DSM-IV for PTSD and depression within adults (Sheet, 2001). Therefore, most EMDR treatments for children exposed to CT before the age of 12 tend to focus on behavior problems (internalizing and externalizing) (Rodenburg et al., 2009). Moreover, as children are in a constant process of change and development across cognitive, neurological, and emotional and relational domains, children with CT have complex needs and require a range of interventions in addition to EMDR (Tufnell, 2005). EMDR should be considered as an adjunct treatment within a multimodal integrative treatment plan, potentially including integrative family therapy (Gold, 2000; Shapiro et al., 2007; Briere and Lanktree, 2008), rather than as the sole or primary treatment for children with CT (Briere and Lanktree, 2008).

Several limitations should be considered when interpreting the conclusions of this systematic review. Firstly, the studies included were very heterogenous, with variable participant characteristics and experiences of trauma as well as variable control groups, length of EMDR treatment, measures of PTSD symptoms, and length of follow-up. This makes interpreting the literature and drawing conclusions a significant challenge. We recommend future investigators to prioritize standardizing the format and length of EMDR treatment, developing a consensus around outcome measures of PTSD symptoms, and replicating findings in equivalent populations and with equivalent comparison groups. Secondly, due to the heterogeneity in comparison in control groups, we were unable to evaluate the overall effect estimates of EMDR vs. other clinical treatments. In the future, a network meta-analysis may be able to evaluate a comparison of EMDR with other single interventions if enough RCTs for CT are available. Thirdly, the individual studies suffered from problems such as drop-out at follow-up, limited treatment length, limited follow-up length, lack of independent assessment of treatment integrity, single therapists for treatment arms and small samples. Therefore, there is a need for well-designed randomized controlled clinical trials of sufficient treatment length and follow-up in the future.

Future studies should also consider some of the real-world challenges in treating people who have experienced complex childhood traumas. Community clinicians often find that a lengthy period of safety and stabilization is required before survivors can actively engage in processing their trauma (McLean et al., 2017) but none of the studies in this review report measuring the nature and length of any such period or investigating its effect on treatment; we therefore recommend that future studies clearly document the prior treatment histories of participants. Furthermore, studies which include use of integrated treatment plans, multidisciplinary interventions and clinician engagements may be more reflective of real world experiences of therapy, including the trust that is built with patients. More broadly, evidence suggests that many symptoms beyond PTSD are more frequently present in patients with CT, including dysregulation of emotions/impulsivity, deficits in attention and consciousness, problems with self-perception, attachment failure, self-destructive behavior, dissociation, interpersonal difficulties and somatization (Kliethermes et al., 2014). Despite the fact that achieving a functional end-state and adaptive psychosocial adjustment is often the secondary target in treating patients with CT (Galovski et al., 2005), few interventional studies for CT include explicit assessment of the above unique symptoms.

In summary, there is growing evidence in both adults and adolescents to support the clinical efficacy of EMDR for reducing symptoms of PTSD (as well as depression and anxiety) associated with complex childhood trauma. However, the small number of trials and the heterogeneity of control conditions, outcome measures and study populations mean we cannot draw firm conclusions; more RCTs are needed to evaluate the efficacy of EMDR in comparison with other psychological interventions and determine which components of therapy drive improvements. Furthermore, studies which address some of the real-world challenges of treating complex childhood trauma will also be important going forward.

## Author contributions

RC: protocol design, search, screening and assessment of studies, data extraction, quality assessment, data analysis and interpretation, writing the article; YZ: search, screening, and assessment of studies, data extraction, quality assessment, data analysis and interpretation, writing the article; AG: data analysis and interpretation, writing the article; YX: supervision of the review, writing, and reviewing the final article; YR: writing and reviewing the final article; LM: protocol design, supervision of the review, writing, and reviewing the final article. All authors read and approved the final manuscript.

### Conflict of interest statement

The authors declare that the research was conducted in the absence of any commercial or financial relationships that could be construed as a potential conflict of interest.

## Footnotes

^1^The Francine Shapiro Library search results (total: 3,529) were screened separately due to lack of reference export capabilities. Subsequently, these results were not included in the numbers for the other electronic databases due to the significant unquantified duplication.
